# Enhanced performance of nanocomposite membrane developed on sulfonated poly (1, 4-phenylene ether-ether-sulfone) with zeolite imidazole frameworks for fuel cell application

**DOI:** 10.1038/s41598-023-34953-8

**Published:** 2023-05-22

**Authors:** Bita Soleimani, Ali Haghighi Asl, Behnam Khoshandam, Khadijeh Hooshyari

**Affiliations:** 1 Faculty of Chemical, Petroleum and Gas Engineering, Semnan University, Semnan, Iran; 2 Faculty of Chemistry, Department of Applied Chemistry, Urmia University, Urmia, Iran

**Keywords:** Environmental sciences, Chemistry, Engineering, Materials science

## Abstract

Proton exchange membrane fuel cells (PEMFC) have received a lot of interest and use metal–organic frameworks (MOF)/polymer nanocomposite membranes. Zeolite imidazole framework-90 (ZIF-90) was employed as an addition in the sulfonated poly (1, 4-phenylene ether-ether-sulfone) (SPEES) matrix in order to investigate the proton conductivity in a novel nanocomposite membrane made of SPEES/ ZIF. The high porosity, free surface, and presence of the aldehyde group in the ZIF-90 nanostructure have a substantial impact on enhancing the mechanical, chemical, thermal, and proton conductivity capabilities of the SPEES/ZIF-90 nanocomposite membranes. The results indicate that the utilization of SPEES/ZIF-90 nanocomposite membranes with 3wt% ZIF-90 resulted in enhanced proton conductivity of up to 160 mS/cm at 90 °C and 98% relative humidity (RH). This is a significant improvement compared to the SPEES membrane which exhibited a proton conductivity of 55 mS/cm under the same conditions, indicating a 1.9-fold increase in performance. Furthermore, the SPEES/ZIF-90/3 membrane exhibited a remarkable 79% improvement in maximum power density, achieving a value of 0.52 W/cm^2^ at 0.5 V and 98% RH, which is 79% higher than that of the pristine SPEES membrane.

## Introduction

The adverse impact of the widespread use of fossil fuels on the environment, specifically with respect to climate change, has resulted in significant efforts to identify and implement feasible and sustainable alternatives. As a result, there is an increasing focus on exploring and utilizing environmentally-friendly renewable energy sources, including hydrogen. One of the energy production systems that utilizes hydrogen fuel is fuel cells^1^. Researchers have taken an interest in the Proton Exchange Membrane Fuel Cell (PEMFC) as a green energy technology among various fuel cells, owing to its distinctive features and benefits. These advantages include high start-up speed, efficiency, and current density, along with a low operating temperature and emission-free operation^2^. Actually, one of the most essential parts of PEMFCs is the proton exchange membrane, which directly determines whether the fuel cell performs successfully or not. Therefore, preparing a suitable membrane for application and accelerating the commercialization process in PEMFC has been one of the main goals of many researchers^3^. A number of non-flourinated polymers, such as sulfonated poly (ether ether ketone)^4^, sulfonated poly (phthalazinone ether ketone)^5,6^, poly vinyl alcohol^7^, and sulfonated poly ether sulfone^8–10^, have recently been investigated as alternatives to commercial Nafion. A new family of coordination polymers known as metal–organic frameworks (MOFs) has been identified that is made up of metal clusters attached to organic ligands that have a three-dimensional crystalline structure^11^. MOFs have various applications such as storage, separation, and catalysis and are also used as biological carriers in medicine^12–15^. Among the various applications, a large number of MOFs have shown good potential for proton and ion conduction^16–18^. MOFs have a high proton conductivity due to their highly flexible design, free surface, and high porosity^11,19^. The ZIF belongs to the large family of MOFs and is made by connecting a divalent metal ion (often Zn^2+^) to four imidazole anionic linkers. It has characteristics like a very high surface area, great thermal and chemical stability, and a flexible and controllable structure^20,21^. The imidazole ring’s presence, according to Zhang group^22^, increased proton conductivity.

Therefore, nanocomposite membranes, which are a combination of MOFs and polymers, are one of the bright perspectives in PEMFC^11^; because the good properties of MOFs incorporated in the polymer lead to the production of new nanocomposite membranes. Numerous reports on the production of new nanocomposite membranes that combine polymer and various MOFs such as ZIF-8^23–26^, UIO-66^27,28^, HKUST-1^29^, CPO-27-Mg^30^, MIL -53-Al^30^, MIL-101 (Cr)^31,32^, and MOF-808^33^, have been performed.

For instance, SPEEK/sulfonated-MIL-101 (Cr) composite membranes were constructed by Li et al.^32^. When compared to a pure SPEEK membrane with a conductivity of 156 mS/cm at 75 °C and 100% relative humidity (RH), the research findings revealed that the newly developed composite membrane exhibited a significantly higher proton conductivity of 306 mS/cm at the same temperature and humidity conditions, which represents an increase of 96.2%. Maiti et al.^34^ utilized molecular dynamics simulations to investigate the potential advantages of incorporating Propylsulfonic acid-functionalized graphene oxide (PrSGO) into a blend of SPEEK and sulfonated poly(benzimidazole) (SPBI) to enhance several material properties, including glass transition temperature (Tg), mechanical strength, proton conductivity, and fuel cell performance. Notably, the XSPEEK/SPBI/PrSGO nanocomposite membrane containing 4 wt. % PrSGO showed a significant increase in proton conductivity, achieving a value of 170 mS/cm at 100% RH and 90 °C. The proton conductivity of the novel ZIF-8@geraphen oxide (GO)/Nafion nanocomposite membranes was measured by Yang et al.^35^. They discovered that the novel membrane's proton conductivity was 280 mS/cm at 120 °C and 40% RH. The SPEEK/ZIF-8/carbon nanotube(CNT) (ZCN) nanocomposite membranes were studied by Sun et al.^24^. At 120 °C and 30% RH, the SPEEK/ZCN-2.5 nanocomposite membrane's proton conductivity was 50 mS/cm. In a different report, Wu et al.^27^, combined S-UiO-66@GO with SPEEK. They discovered that at 70 °C (95% RH) and 100 °C (40% RH), respectively, the proton conductivity of the SPEEK/S-UiO-66@GO-10 composite membrane obtained 268 mS/cm and 165.7 mS/cm. In their study, Kim et al.^36^ investigated the potential of using phenylsulfonic-acid functionalized and unzipped graphite nanofiber (SO_3_H-UGNF) to develop a nanohybrid membrane by incorporating it with SPEEK for a PEFC operating under low RH conditions. Their findings revealed an optimized SPEEK/SO_3_H-UGNF (1 wt%) nanohybrid membrane that exhibited improved properties such as excellent proton conductivity, increased power density, and greater durability when compared to the SPEEK membrane. Vinothkannan et al.^37^ The study presents a hybrid membrane architecture composed of poly arylene propane biphenyl (FPAPB) and SPEEK blended with Iron oxide (Fe_3_O_4_) anchored functionalized graphene oxide (Fe_3_O_4_-FGO), which improves proton conductivity, water absorption, and ion exchange capacity while maintaining dimensional stability. The peak proton conductivity of the aligned quadratic hybrid membrane is 11.13 mS/cm at 120 °C and 20% RH, outperforming the pristine SP membrane and Nafion-112 membrane while exhibiting lower gas permeability.

In a separate study, Rao et al.^38^ fabricated composite membranes comprised of UIO-66-NH_2_@GO/Nafion. Their research demonstrated that the proton conductivity of these membranes reached 303 mS/cm when tested under conditions of 90 °C and 95% relative humidity. Barjola et al.^24^, conducted measurements to determine the conductivity of protons in novel membranes such as SPEEK/ZMix, (ZMix is made by combining ZIF-7 and ZIF-8), SPEEK/Z8 (ZIF-8), and SPEEK/Z7 (ZIF-7). The results of their study indicated that at a temperature of 120 °C, the proton conductivity for these new membranes was reported to be 8.5 mS/cm, 2.5 mS/cm, and 1.6 mS/cm, respectively. Zhang et al.^39^, have developed new composite membranes composed of sulfonated poly arylene ether ketones (SPAEKs) and Imidazole-MOF-801 (Im-MOF-801). These membranes exhibit high proton conductivity, with a value of 128 mS/cm at 90 °C and 100% RH. Notably, the composite membrane's proton conductivity significantly surpassed that of SPAEKs polymer operating under identical conditions. Duan et al.^40^ developed the use of a bi-functionalized MOF based on amino-sulfonic acid, along with a sulfonate nano fiber (SNF)- PAEK membrane. The modification method employed in the study was a one-step process. The results showed that the MNCS@SNF-PAEK-1.5 membrane exhibited the highest proton conductivity of 188 mS/cm, which holds great promise in improving the performance of PEMs by utilizing the MOFs and sulfonated polymers.

Compared to other composite membranes composed of ZIFs, ZIF-90 demonstrates an exceptional level of chemical flexibility, primarily attributed to the presence of an aldehyde group. This functional group plays a crucial role in enhancing the membrane's water retention capability, resulting in remarkable performance characteristics, such as superior thermal and chemical stability, heightened proton conductivity, and increased water uptake. Consequently, ZIF-90 surpasses previously published ZIF-8 and ZIF-7 membranes in these aspects^41,42^. Sulfonated poly (1, 4-phenylene ether-ether-sulfone) (SPEES) is a sulfonated aromatic polymer that exhibits robust mechanical, thermal, and chemical stability, while being relatively cost-effective to produce^43–46^. Despite the numerous features of the SPEES membrane, its proton conductivity is currently insufficient to achieve the desired efficiency for PEMFCs. Consequently, there has been a significant focus on addressing these limitations and improving proton conduction in PEMFCs through various efforts and developments.

In this paper, with the goal of improving the conductivity of proton, the properties of the SPEES membrane with ZIF-90 nanostructure have been modified. In order to, the first step, ZIF-90 was synthesized. So, the different amount of made ZIF-90 was added to SPEES membranes. The final step involved measuring a number of characteristics, including water uptake, proton conduction, and fuel cell performance.

## Experimental

### Materials

All materials are bought from Sigma Aldrich and Merck and used in the same purity. Poly (1, 4-phenylene ether-ether-sulfone) (PEES) and 2-Imidazole carboxyldehyde (ICA) were provided by Sigma-Aldrich. Trioctylamine (TOA), Zinc nitrate (Zn (NO_3_)_2_.6H_2_O), ethanol, concentrated sulfuric acid (purity, ˃ 98%), dimethyl acetamide (DMA) and dimethyl formaldehyde (DMF) were purchased from Mercke company.

### Synthesize of ZIF-90

The ZIF-90 nanostructure has been synthesized according to the procedure^47^. In summary, in this method, 0.75 mmol of zinc nitrate cluster and 2.10 mmol of 2-imidazole carboxyhydride linker are solved separately in 50 mL and 100 mL of DMF, respectively. In the third step, 1.96 ml of trioctylamine is dissolved separately in 50 mL of DMF solvent at ambient temperature. So the zinc nitrate metal cluster is slowly added to the ICA organic linker. In the final step, trioctylamine is added to the solution. Finally, the product is centrifuged and after washing for several with ethanol solvent and the end is dried in a vacuum oven at 80 °C for 12 h.

### Sulfonation of PEES

**Figure 1:**
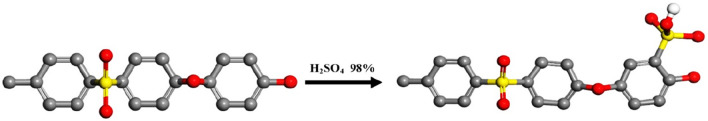
Schematic of PEES sulfonation. (White: Hydrogen, Yellow: Sulfur, Red: Oxygen, Gray: Carbon).

According to the reference, SPEES was obtained through the postsulfonation of PEES (Fig. 1)^48^. In briefly 20 mL of 98% concentrated sulfuric acid, 2 g of PEES polymer is dissolved at room temperature. After 12 h at 25 °C, the solution dissolves on a magnetic stirrer. Then, for extracting the sulfonated polymer, uniform solution is added slowly and dropwise to cold deionized water (containing ice). This action results in the precipitation of the sulfonated polymer. The produced polymer is washed with deionized water to neutralize the pH (pH = 7). The produced polymer is dried in a vacuum oven at 100 °C. The titration method was used to determine the sulfonation degree (DS) of SPEES in this work. The DS was calculated to be around 68%.

### Construction of nanocomposite membranes

Solution-casting was used to produce the nanocomposite membranes. Proton exchange composite membranes have been used in a variety of works using the solution casting approach^43,49,50^. First, to create a perfectly homogeneous yellow solution, 0.2 g of SPEES polymer is dissolved in 2 mL of DMAc solvent at 60 °C and placed on a magnetic stirrer. The mixture of different percentages of nano-ZIF-90 (0.5–7 wt%) in 1 mL DMAc is spread by ultrasonic for 30 min. The above solution containing ZIF-90 nanoparticles is added to the yellow solution containing SPEES and placed on a magnetic stirrer for 4 h until completely homogeneous. The prepared solution is poured on a petri dish and dried in a multi-step process. It is dried in an oven at 80 °C for 24 h to evaporate the solvent and create a uniform dry polymer film after being first placed at room temperature for 24 h. In final for several steps, it is rinsed in deionized (DI) water to remove excess solvent. The SPEES/ZIF-90/x nanocomposite membranes with x: 0.5 wt. %, 1 wt. %, 2 wt. %, 3 wt. %, 4 wt. %, 5 wt. % and, 7 wt. % loading of ZIF-90 are marked as SPEES/ZIF-90/0.5, SPEES/ZIF-90/1, SPEES/ZIF-90/2, SPEES/ZIF-90/3, SPEES/ZIF-90/4, SPEES/ZIF-90/5 and SPEES/ZIF-90/7 respectively. The membranes thickness were at around 70 µm.

### Characterization

The ZIF-90 nanostructure’s successful synthesis was confirmed by FT-IR, XRD, and N_2_ adsorption analyses. The BELSORP MINI II adsorption instrument manufactured by Microtrac (Japan) measured the Langmuir surface area, specific Brunauer–Emmett–Teller (BET), pore volume, and pore size distribution. The 8400S model was subjected to Fourier transform infrared spectroscopy (FTIR) analysis (Germany). The X-ray diffraction (XRD) analysis was conducted using the Bruker D8 and GNR Explorer diffractometers from Italy, utilizing Cu Kα radiation. With a resolution of 4 cm^−1^ and a region of 600–4000 cm^−1^, Bruker Equinox 55 was used to perform the ATR-FTIR spectra. The morphology of the SPEES/ZIF-90 nanoocomposite membranes was seen using a TESCAN MIRA 3 field emission scanning electron microscope (FESEM). The morphology-phase atomic force microscopy (AFM) JPK NanoWizard II model manufactured by BRUKER was utilized to examine the membrane morphology. On a LINSEIS, analyses using thermogravimetric analysis (TGA) were carried out under atmosphere at a heating rate of 10 °C/min. DSC analyses were obtained using the Q600 (USA) at a rate of 10 °C/min in a N_2_ atmosphere. Mechanical parameters of the dry membranes were used by Santam STM-50 model with the velocity of 10 mm.min^−1^. Using a potentiostat–galvanostat Metrohm called the PGSTAT303N, proton conductivity measurements were performed. The conductivity of proton (σ) was obtained from the following relation^50^:

1 $$\sigma =\frac{L}{RS},$$

Here L represents the membrane thickness (cm), R is the resistance obtained from the Nyquist curve (ohm), and S is the membrane surface area (cm^2^).

The slope of the Arrhenius plots can be operated to determine the Activation energy (E_a_) by following relation:

2 $$6=A\,\mathrm{exp}\left(-\frac{{E}_{a}}{RT}\right),$$

Here, A is the Arrhenius constant, R is gas constant (8.314 J/mol.K) and T was the temperature (Kelvin).

The water uptake (WU)) is obtained from the difference between dry (W_dry_) and wet weight (W_wet_) (after 24 h of immersion in water) of the membrane from Eq. (3) that using the method reported in references^50,51^.

3 $$WU \left(\%\right)=\frac{{W}_{wet}-{W}_{dry}}{{W}_{dry}}\times 100.$$

The IEC value of the membrane was deter defined mined by the conventional titration method as reported elsewhere^49,50^.

4 $$\mathrm{IEC }\left(\frac{\mathrm{meq}}{\mathrm{g}}\right)=\frac{{V}_{NaOH}\times {M}_{NaOH}}{{W}_{M}},$$

where M_NaOH_ was the molar concentration of NaOH solution (0.1 M), V_NaOH_ was the volume of NaOH solution (L) and W_M_ was the weight of a dry sulfonated polymer (SPEES (g)). Degree of SPEES sulfonation depends on the IEC and is described by the following relation^50^.

5 $$\mathrm{DS }\left(\mathrm{\%}\right)=\frac{324\times IEC\times 100}{(1000-102\times IEC)},$$

For investigation the oxidation stability of membranes, Fenton test was done based on the procedure explained by Grot and LeClech^52,53^. The weight loss percentage in membrane can be calculated according to:

6 $$\mathrm{WL }\left(\mathrm{\%}\right)=\left(\frac{\mathrm{W}0-\mathrm{W}1}{W0}\right)\times 100.$$

The creation of membrane electrode assemblies (MEAs) is necessary to investigate the PEMFC's final performance. The catalyst ink is first prepared by dissolving the specified quantity of 20 wt. % Pt-C powder in isopropyl alcohol/water and a SPEES solution. A carbon fiber fabric with a microporous layer and a loading of 0.5 mg/cm^2^ will be painted with catalyst ink. The second step involves drying the prepared electrodes between 80 °C and 120 °C. To create the electrode-membrane assembly, the prepared electrodes and membrane were squeezed at 50 kg/cm^2^ for 5 min at 120 °C. Finally, the potential was held constant at 0.5 V for 6 h until the temperature reached 80 °C in order to activate the produced MEAs. Finally at flow rates 300/500 mL/min of hydrogen/Oxygen were inserted into the anode and cathode electrodes.

## Results and discussions

### Characterization of ZIF-90

**Figure 2:**
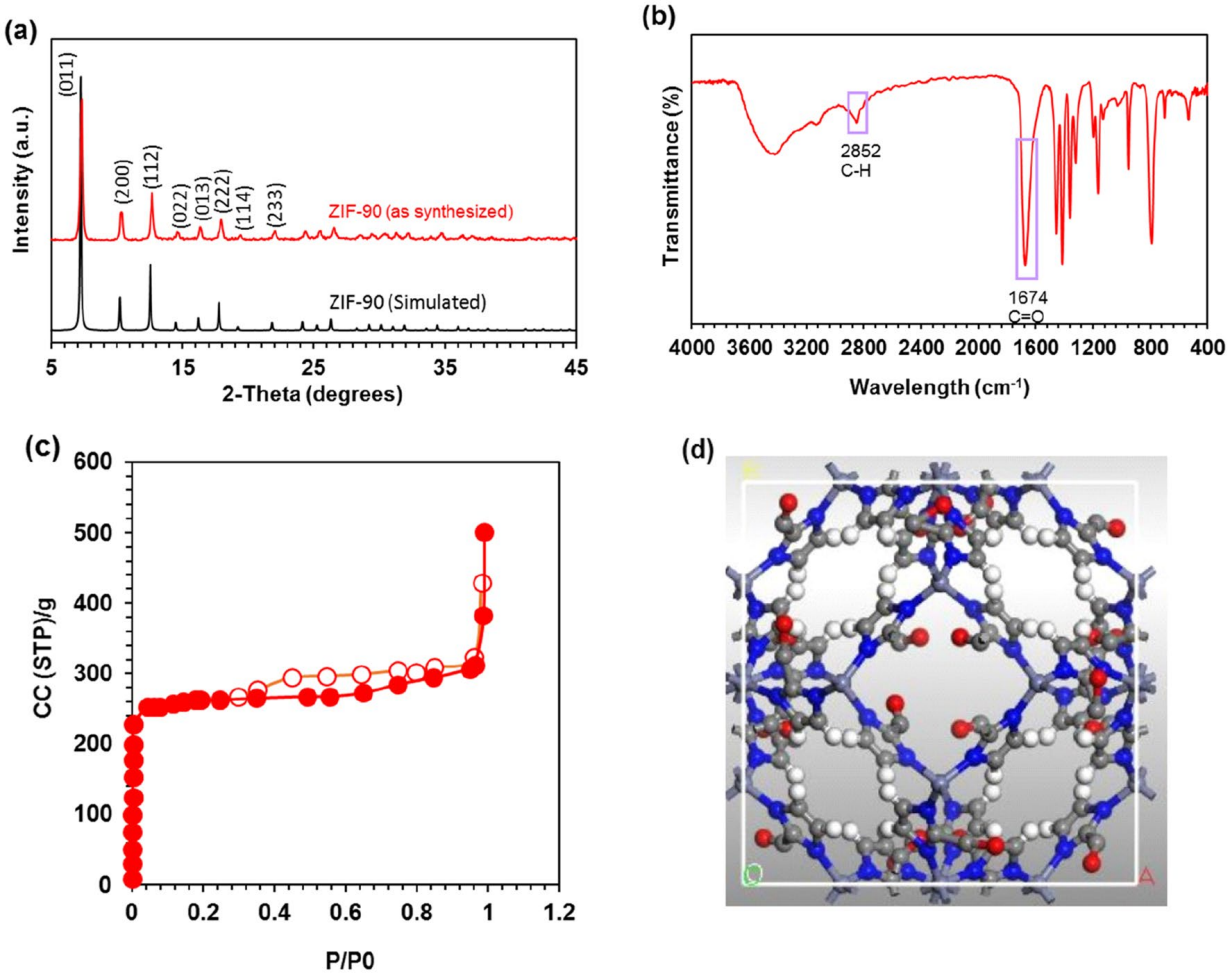
(**a**) XRD pattern, (**b**) FT-IR spectra of synthesized ZIF-90, (**c**) N_2_ adsorption (filled marks) and desorption isotherm (blank marks) at 77 k for ZIF-90, (**d**) Crystal structure of ZIF-90. (Purple: Zinc, White: Hydrogen, Blue: Nitrogen, Red: Oxygen, Gray: Carbon).

Figure 2a displays the X-ray diffraction (XRD) pattern of ZIF-90. The prominent XRD peaks of the ZIF-90 structures are completely set with the standard patterns learned from simulations expressing their successful syntheses, as shown in Fig. 2a. The pattern of peaks observed at 2θ = 7.28°, 10.46°, 12.74°, 15.08°, 16.46°, 18.08°, 19.64°, and 22.28°, corresponding to the intensities of (011), (200), (112), (022), (013), (222), (114), and (233) crystallographic planes, respectively, agrees with the single crystal data of simulated ZIF-90. The crystal structure of ZIF-90 has been successfully formed, according to the XRD pattern.

As shown in Fig. 2b, the purity and bonding characteristics of the ZIF-90 structure produced using the FT-IR spectrum are examined. The peaks at 3417 cm^−1^ and 3282 cm^−1^ in Fig. 2b are connected to the aromatic stretching vibration’s N–H and C–H bonds. The peaks in the region of 1674 cm^−1^ and 2852 cm^−1^ are the tensile vibrations of the C = O aldehyde group and the C–H in the aldehyde group, respectively. While the peaks in the region of 1361 cm^−1^, 1415 cm^−1^, and 1456 cm^−1^ are related to the C–H, C = C, and C = N flexural vibration of the ring, respectively, the peaks located in the 600–1500 cm^−1^ region are related to the total tensile or flexural vibrations of the imidazole ring. These peaks confirm the ZIF-90 structure, which is in line with earlier studies^11^.

The nitrogen adsorption and desorption isotherm at − 196 °C (77 K) is depicted in Fig. 2c. Additionally, the measured ZIF-90 nanostructure properties are compiled in Table 1 and include the BET contact surface, pore volume, and pore diameter. The present study reports a measured BET surface area of 1180 m^2^/g for ZIF-90. The adsorption/desorption isotherms exhibit a classification of Type I according to IUPAC standards. This indicates that the primary pores of the adsorbent substance fall within the micro range. A review of the data demonstrates that ZIF-90's N_2_ adsorption/desorption isotherm accurately reveals the structure of the sample that was synthesized using the available sources^11,19^. The crystal structure of ZIF-90 is also displayed in Fig. 2d ZIF-90 (as synthesized). The Crystallographic Cambridge Data Centre (CCDC) offers access to the Crystallographic Information Files (CIFs) for the structure of ZIF-90 (https://www.ccdc.cam.ac.uk/).

**Table 1:** Properties of the synthesized ZIF-90.

BET surface area (m^2^/g)	Langmuir surface area (m^2^/g)	Mean pore diameter (nm)	Total pore volume (cc/g)*
1180	1270	3.52	0.568

*Calculated at P/P_0_ = 0.99.

### Physicochemical properties of the SPEES/ZIF-90/x nanocomposite membrane

**Figure 3:**
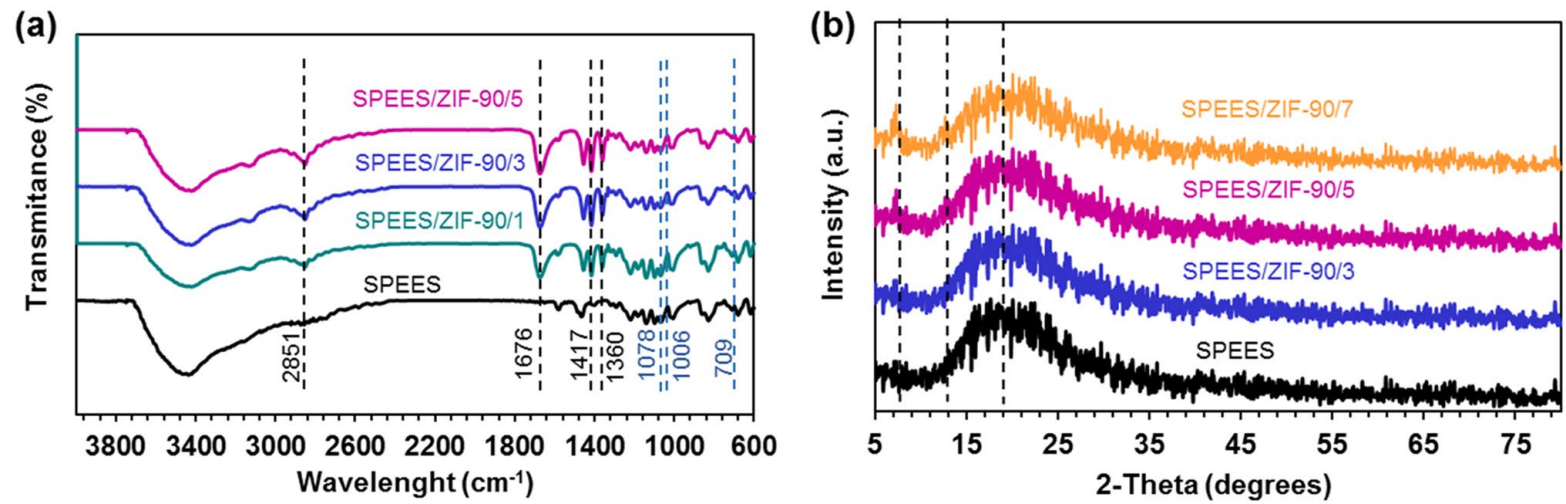
(**a**) ATR-FTIR spectra, (**b**) XRD of SPEES/ZIF-90/x nanocomposite membrane.

Figure 3a depicts the bonding and structure nature of the SPEES/ZIF-90/x nanocomposite membranes produced with the ATR-FT-IR spectrum. According to Fig. 3a, the peak located at 3420–3430 cm^−1^ corresponds to the tensile vibrations of the O–H bond of the –SO_3_H group in the SPEES membrane. The peak located in the 2851 cm^−1^ region corresponds to the C-H tensile vibrations of the aldehyde group and the peak located in 1676 cm^−1^ corresponds to the tensile vibrations of the C = O bond in the aldehyde group of the ZIF-90. The peaks located in the area 1360 cm^−1^ and 1417 cm^−1^ are related to the bending vibrations of C-H and C = C of the imidazole ring. The peaks at 709 cm^−1^, 1006 cm^−1^ and 1078 cm^−1^ correspond to the S–O, O = S = O bond, respectively. The presence of these peaks indicates the formation and approval of the ZIF-90 structure in the SPEES/ZIF-90 nanocomposite membranes with different percentages of ZIF-90^47,50^.

In Fig. 3b indicates the X-ray diffraction pattern of the SPEES, SPEES/ZIF-90/3, SPEES/ZIF-90/5 and SPEES/ZIF-90/7 membranes. The broad crystalline peak in the XRD pattern is visible in the 2θ = 19° (related to the SO_3_H group) for the SPEES membrane, which corresponds to the relevant reference^54^. As shown in the Fig. 3b, the broad peak is visible in all membranes. The intensity of peak width is reduced by increasing the ZIF-90 content in SPEES/ZIF-90/x nanocomposite membranes. This may be due to the presence and effect of ZIF-90 nanostructure on SPEES membranes. On the other hand, the presence of ZIF-90 in SPEES/ZIF-90/x nanocomposite membranes with 2θ = 7° and 2θ = 12° peaks has been shown^47^.

**Figure 4:**
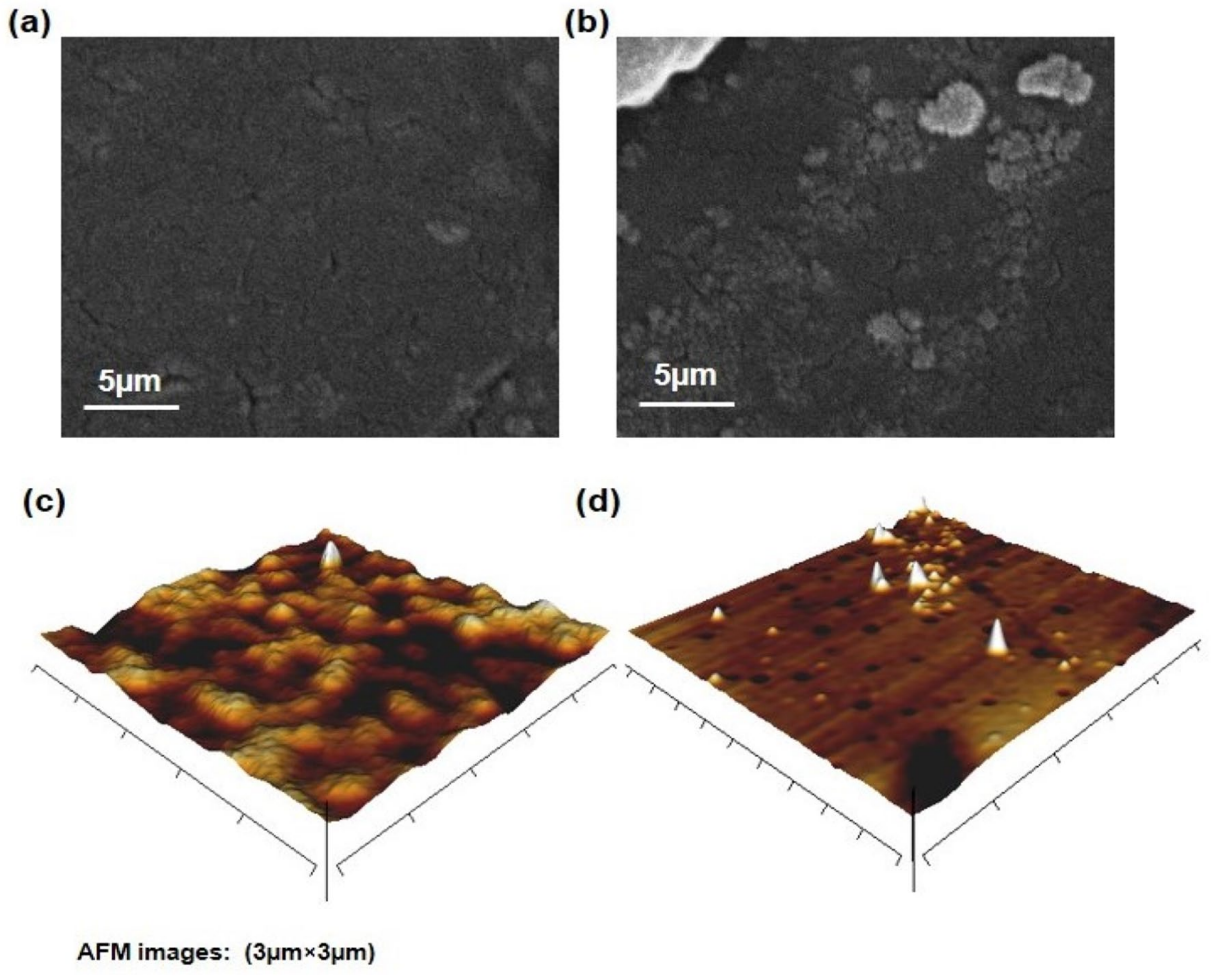
FESEM-AFM image of cross-section of the (**a,c**) SPEES/ZIF-90/3,and (**b,d**) SPEES/ZIF-90/5membranes.

In Fig. 4, exhibits the cross-sectional images of the FESEM-AFM corresponding to the SPEES/ZIF-90/3 and SPEES/ZIF-90/5 membranes. Figure 4a shows the FESEM image of the SPEES/ZIF-90/3 nanocomposite membrane, which shows the uniform distribution of ZIF-90 on the basic membrane. The cross-section of the SPEES/ZIF-90/3 has suitable morphology. Figure 4b shows the accumulation of ZIF-90 nanostructure on the surface of SPEES/ZIF-90/5 nanocomposite membrane with 5 wt. % of ZIF-90. Figure 4c,d presents the AFM surface image of the SPEES/ZIF-90/3 and SPEES/ZIF-90/5 nanocomposite membranes. The lighter regions on the image correspond to the hydrophilic groups, while the darker regions correspond to the hydrophobic parts of the membrane. The nanocomposite membranes demonstrate a homogeneous distribution of the ionic channels on the lighter regions. The bright spots observed in the SPEES/ZIF-90/3 membrane, as depicted in Fig. 4c, suggest that the membrane possesses desirable hydrophilic properties.

### Thermal, chemical and mechanical properties of nanocomposite membranes

**Figure 5:**
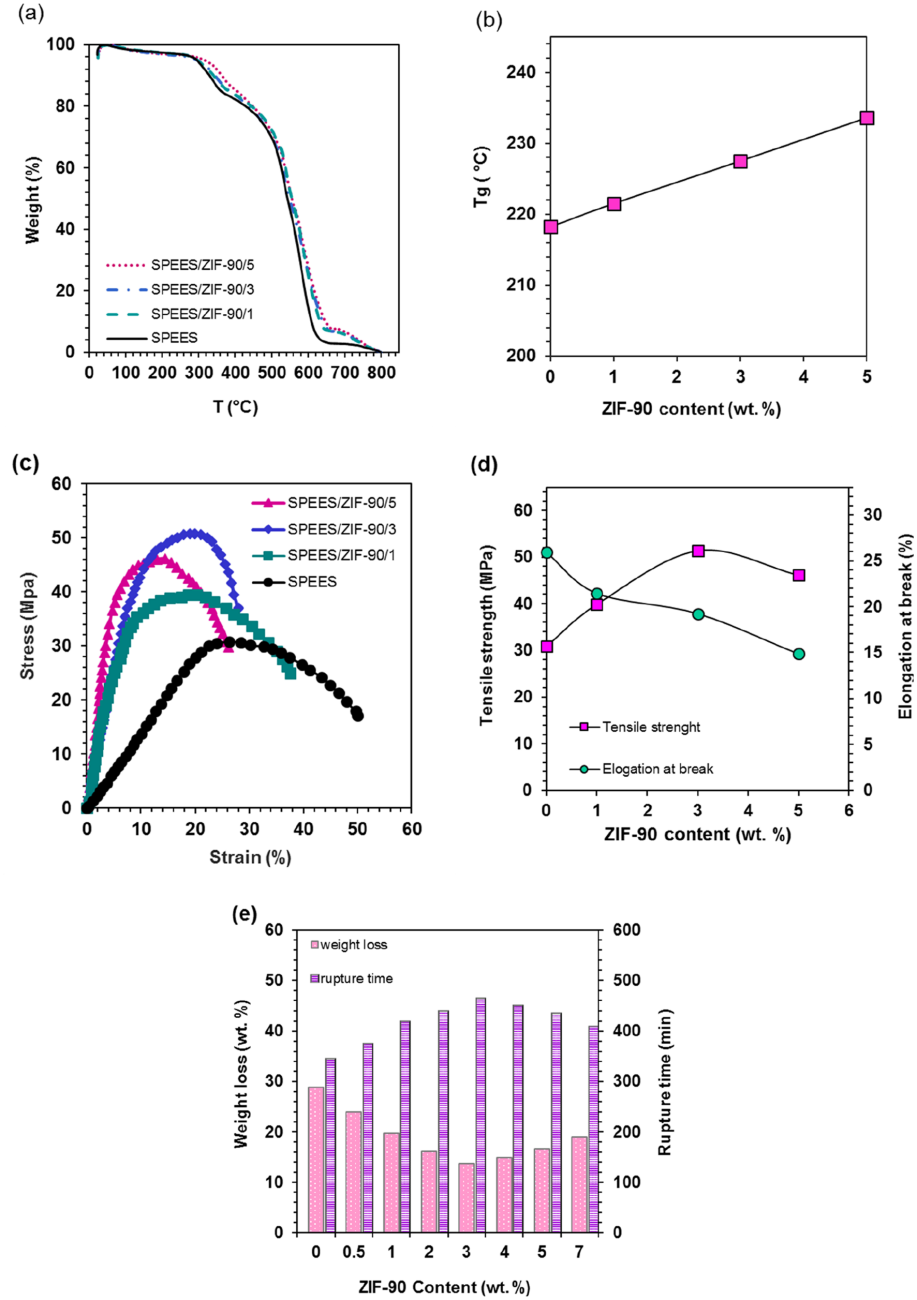
(**a**) TGA, (**b**) T_g_ results of SPEES, SPEES/ZIF-90/1, SPEES/ZIF-90/3 and SPEES/ZIF-90/5 membranes, (**c**) Stress–Strain curve, (**d**) The trend of changing the maximum tensile strength applied and Elongation at break in different membranes, (**e**) Chemical stability of nanocomposite membranes.

In the Fig. 5a displays the TGA of the SPEES, SPEES/ZIF-90/1, SPEES/ZIF-90/3 and SPEES/ZIF-90/5 membranes. The breakdown of the SO_3_H functional group is what causes the first weight loss in the 290–370 °C temperature range^24,50,55^. Due to the main polymer chains degrading, the second weight loss occurred at a temperature of about 480 °C. With the presence of ZIF-90 in nanocomposite membranes, the intensity of temperature decrease slope is reduced. All membranes produced up to 290 °C have thermal stability. Also in the Fig. 5b shows the trend of T_g_ changes of the SPEES, SPEES/ZIF-90/1, SPEES/ZIF-90/3 and SPEES/ZIF-90/5 membranes. The T_g_ in the SPEES membrane is reported about 218.2 °C^50^. The glass temperatures of the SPEES/ZIF-90/1, SPEES/ZIF-90/3 and SPEES/ZIF-90/5 nanocomposite membranes are 212.5 °C, 227.5 °C and 233.6 °C respectively. With increasing percentage of ZIF-90, the amount of T_g_ has increased.

The stress–strain relationship between the membranes for SPEES, SPEES/ZIF-90/1, SPEES/ZIF-90/3, and SPEES/ZIF-90/5 is shown in Fig. 5c. The maximum applied tensile strength and the elongation at break for various membranes are also shown in Fig. 5d. The curves show that the force applied to the SPEES/ZIF-90/3 membrane, with a value of 51.385 MPa, results in the greatest resistance. With more ZIF-90 present, however, the amount of elongation decreases. These findings demonstrate how the addition of ZIF-90 can significantly enhance the thermal, chemical, and mechanical characteristics of nanocomposite membranes.

Differences in chemical stability of different membranes are indicated in Fig. 5e. The results indicate that the rupture time and weight loss versus increasing percentage of ZIF-90. For a 3 wt. % of ZIF-90, the weight lost relative to the SPEES polymer membrane is halved and the rupture time is increased by 2 h, and the claim of increased chemical stability can be proved by the presence of 3 wt. % ZIF-90. Increasing the values by more than 5 wt. % ZIF-90 reduces the chemical stability that may be due to the accumulation of ZIF-90.

### Proton conductivity of nanocomposite membranes

The properties of the SPEES membrane and the SPEES/ZIF-90/x nanocomposite membranes were compared in the WU, IEC and proton conductivity.

As shown in Table 2, with increasing the ZIF-90 content to 3 wt. %, the amount of water uptake has increased from 38.61% to 68.79% at the 25 °C and the other tempratures. So that SPEES/ZIF-90/3 nanocomposite membranes is reported with the highest amount of water uptake in the different temprature. In fact, high porosity and surface area and existing aldehyde group of ZIF-90 is caused to trap water molecule in the pores. The reduction in the percentage of water uptake can be attributed to the accumulation of ZIF-90, as evidenced by the increase in its concentration by over 3 wt. %. The IEC of a membrane shows how many acid groups there are in every gram of the sample and how many ionizable functional groups are present in the membrane. According to Table 2, with the increase in ZIF-90 content by 7 wt. %, the IEC has decreased from 1.73 meq/g to 1.589 meq/g. This decrease is due to enhanicng the presence of ZIF-90 nanostructure and reduction of SO_3_H groups and increasing of electrostatic interactions between the polymer acidic group and the ZIF-90 functional group (aldehyde group)^56–58^.

**Table 2:** WU and IEC of the nanocomposite membranes.

Membrane	IEC (meq/g)	WU (wt. %)			
		25 °C	40 °C	60 °C	80 °C
SPEES	1.73	38.61	48.46	56.1	61.4
SPEES/ZIF-90/0.5	1.701	48.93	57.62	65.18	70.38
SPEES/ZIF-90/1	1.68	57.84	67.34	73.96	79.06
SPEES/ZIF-90/2	1.651	63.83	72.76	80	85.1
SPEES/ZIF-90/3	1.621	68.79	77.69	84.9	89.29
SPEES/ZIF-90/4	1.611	64.03	72.73	80.13	85.03
SPEES/ZIF-90/5	1.598	59.35	68.03	75.13	79.93
SPEES/ZIF-90/7	1.589	49.12	57.33	64.45	69.55

**Figure 6:**
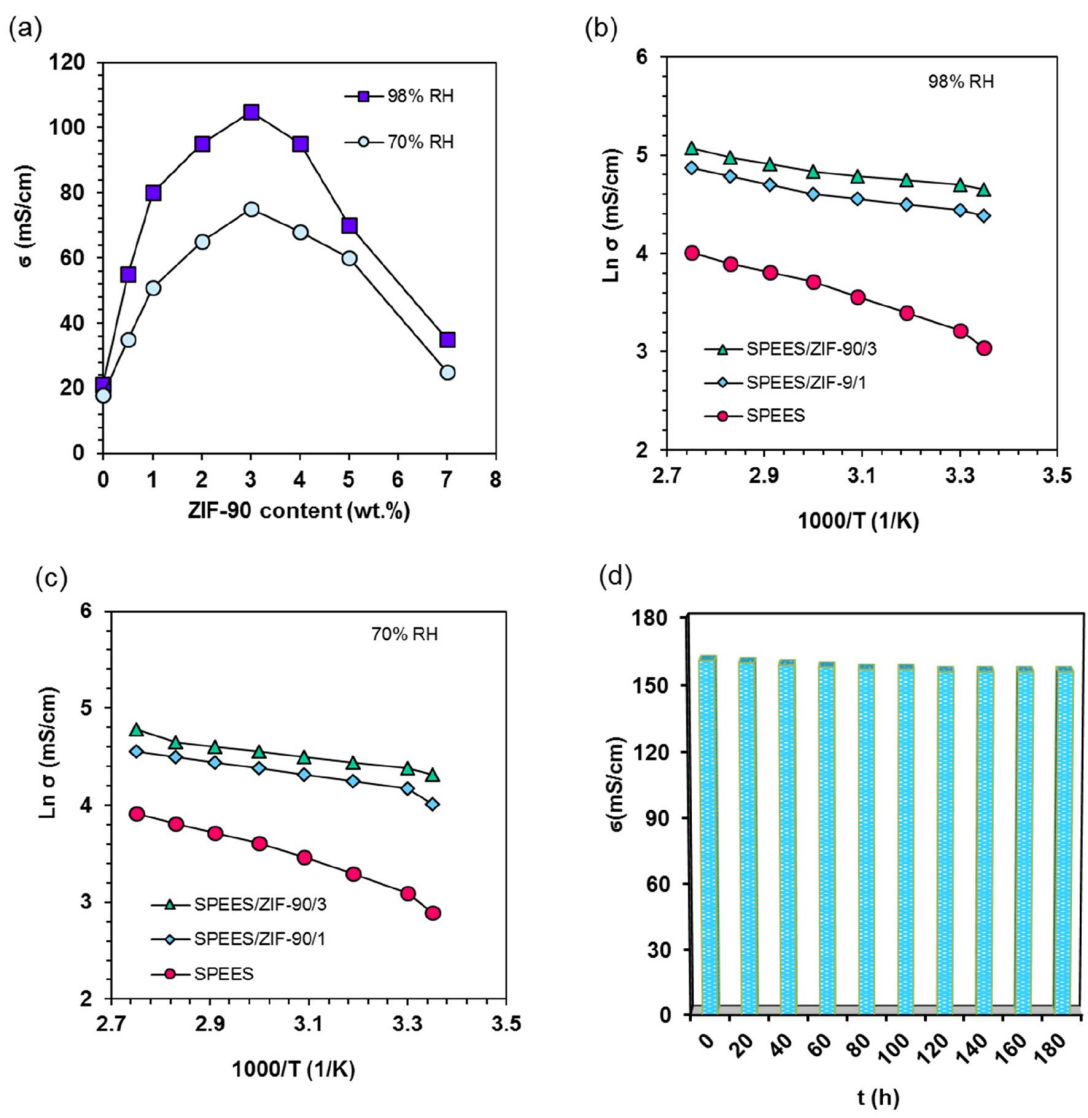
(**a**) Proton conductivity of nanocomposite membranes at 25 °C, (**b**) at different temperature and 98% RH, (**c**) at different temperature 70% RH, (**d**) time stability of SPEES/ZIF-90/3.

The conductivity of Proton is one of the effective parameters for evaluating PEMFC performance. Several elements, including water uptake, IEC, and type of nanoparticles, have an impact on the proton conductivity of nanocomposite membranes. In Fig. 6a shows the proton conductivities of SPEES and their nanocomposite membranes at 25 °C with various percentages of ZIF-90. The proton conductivity of SPEES/ZIF-90/x nanocomposite membranes effectively increases when compared to that of the SPEES membrane, as shown in Fig. 6a. In other words, ZIF-90 is essential for improving the conductivity of protons in nanocomposite membranes. The aldehyde group and imidazole ring also enhance Grotthus' mechanism by facilitating proton transfer at proton hopping sites. Comparing the results, the SPEES/ZIF-90/3 membrane performed better than other membranes with proton conductivities of 105 mS/cm and 75 mS/cm (at 25 °C and 98% and 70% RH, respectively). However, proton conductivity is decreased by blocking proton transport channels at concentrations greater than 5 wt. % ZIF-90. On the other hand, Fig. 6b,c shows the proton conductivity of nanocomposite membranes at various temperatures. The conductivity of protons has increased with temperature because their mobility has improved. SPEES/ZIF-90/3 nanocomposite membranes had conductivities of 105 mS/cm and 160 mS/cm at 25 °C and 90 °C, respectively, according to a comparison of the various nanocomposite membranes. These numbers are greater than the 21 mS/cm and 55 mS/cm proton conductivities of SPEES. This data leads us to assume that the MOFs nanostructure does have a long-term impact on improving proton conductivity on MOF/polymer nanocomposite membranes.

Time-stability is another important parameter in the PEMs. Figure 6d illustrates the proton conductivity lifetime plots of SPEES/ZIF-90/3 membrane at 95 °C and 98% RH. The SPEES/ZIF-90/3 nanocomposite membranes showed stable proton conductivity after 180 h. The SO_3_H group of polymer, -CHO group and imidazole ring of ZIF-90 nanostructure trigger the good hydrogen bonding, trapping the water in the pores and so proton conductivity remains Table.

Table 3 compiles an overview of the literature on Nafion 117 and various sulfonated aromatic polymers' ability to form nanocomposite membranes with proton conductivity. The analysis of the data revealed that the SPEES/ZIF-90/3 nanocomposite membrane’s proton conductivity performed better under the same conditions than the other results mentioned. The increase in water uptake at various temperatures at the membrane's interface, which can lead to stability in the proton transfer pathways, and the even distribution of the ZIF-90 nanostructure are both responsible for this increase.

**Table 3:** Proton conductivity of nanocomposite membrane developed on some sulfonated aromatic polymers and Nafion 117 membrane.

Membranes	Filler loading (wt. %)	T (°C)	RH (%)	Ϭ (mS/cm)	Ref
SPEES/ZIF-90	3	90	98	160	This work
Sulfonated poly(arylene ether sulfone)	–	80	100	146	^59^
Sulfonated poly(arylene ether sulfone)	–	80	100	117	^60^
Sulfonated poly(arylene ether sulfone)s	–	120	100	65	^61^
Sulfonated polysulfone	–	80	100	43	^62^
Sulfonated poly(arylene sulfone)	–	25	100	38	^63^
SPEEK/ZIF-8	2.5	120	30	25	^23^
Sulfonated polysulfone/phosphatoantimonic acid	–	80	100	20	^64^
HKUST-1/Nafion	2.5	25	100	18	^29^
Sulfonated poly(arylene ether sulfone)/ GO	0.5	90	100	17.1	^65^
PSU/sPSU/NH_2_-MIL-53	5	70	100	17	^66^
CPO-27-Mg/Nafion	3	50	100	11	^30^
MIL-53-Al /Nafion	3	50	100	9.8	^30^

### Fuel cell performance

**Figure 7:**
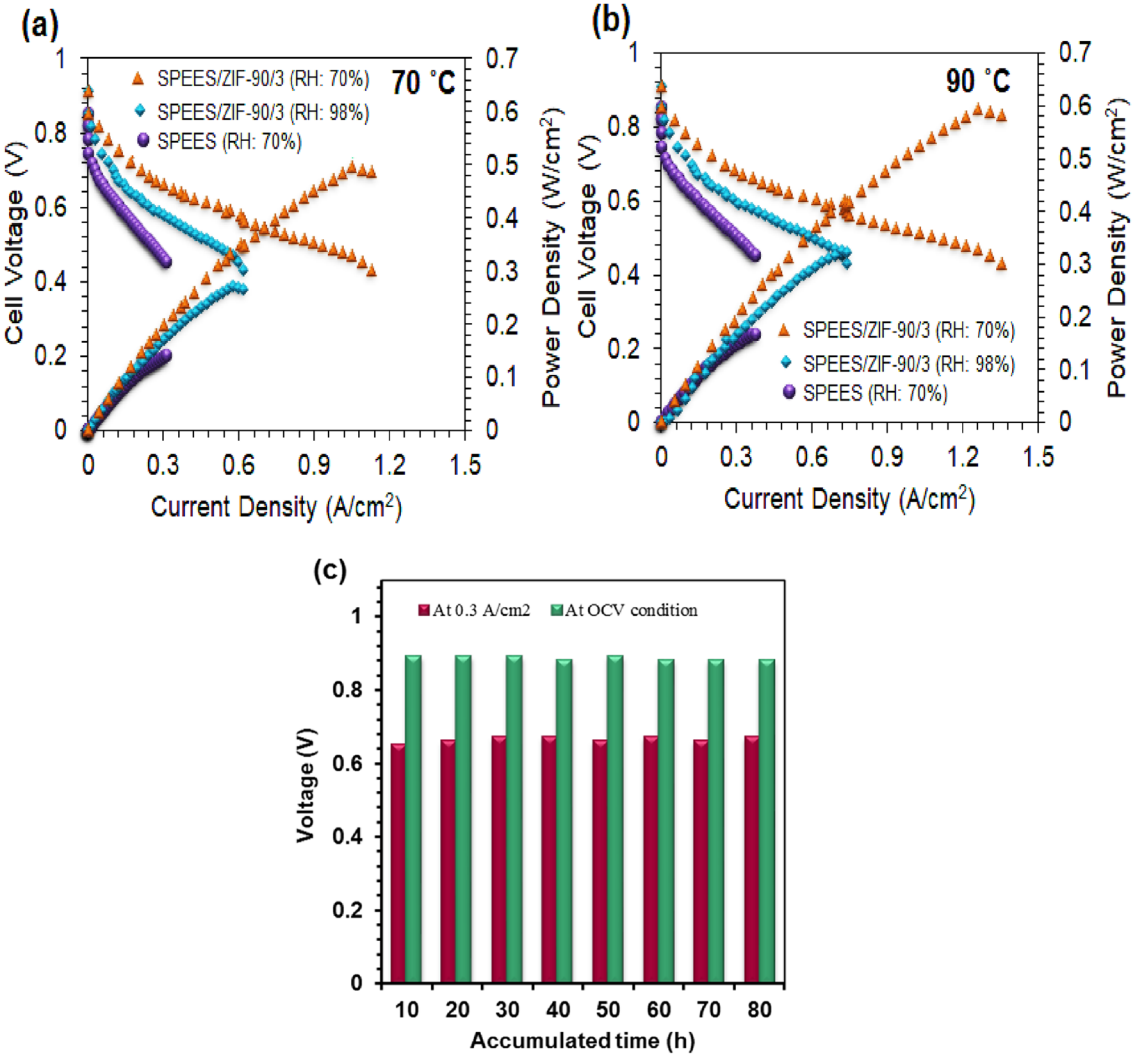
Polarization curves of SPEES and SPEES/ZIF-90/3 membranes at (**a**) 70 °C and (**b**) 90 °C at 70% RH and 98% RH (**c**) Fuel cell life time plots of SPEES/ZIF-90/3 nanocomposite membranes at 90 °C and 98% RH.

As shown in Fig. 7, the current density-potential (I-V) and current density-power density curves of nanocomposite membranes made of SPEES and SPEES/ZIF-90/3 at 70 °C and 90 °C and 70% RH and 98% RH, respectively. The SPEES/ZIF-90/3 membrane’s maximum current densities at 0.5 V, 98% RH, 70 °C, and 90 °C were 0.89 A/cm^2^ and 1.07 A/cm^2^, respectively. According to Fig. 7a,b, the maximum power density of the SPEES/ZIF-90/3 nanocomposite membrane at 90 °C increased from 0.41 W/cm^2^ at 70% RH to 0.52 W/cm^2^ at 98% RH..

The SPEES/ZIF-90/3 nanocomposite membrane (Fig. 7) had the best performance in terms of polarization curves (160 mS/cm at 90 °C and 98% RH), which may be because it is more capable of absorbing water and conducting protons. One of the key elements that affects how well produced membranes perform in the end is proton conductivity, which rises with increasing relative humidity from 70% RH to 98% RH.

Reporting the open circuit voltage (OCV) of the PEMFC for 100 h, as shown in Fig. 7c, allowed for the determination of the long-term stability of the SPEES/ZIF-90/3 nanocomposite membrane at 90 °C and 98% RH. Referring to its high WU (89% at 80 °C) and high mechanical stability, the OCV in the PEMFC constituted by the SPEES/ZIF-90/3 nanocomposite membrane practically maintained a constant quantity after 100 h (tensile strength: 51.385 MPa). The final result was a nanocomposite membrane (SPEES/ZIF-90/3) that performed exceptionally well over an extended period of time.

## Conclusion

One of the intriguing and successful possibilities for improving membranes and boosting the effectiveness of polymer membranes in fuel cell performance is the use of metal organic frameworks (MOFs). In this research, we produced a new Polymer/MOF nanocomposite membrane for use in PEMFC by using this technique. In comparison to a SPEES-based membrane, the SPEES/ZIF-90/3 nanocomposite membrane demonstrated superior proton conductivity of up to 160 mS/cm under 90 °C and 98% RH. This enhanced conductivity is believed to be due to the membrane’s effective water uptake properties, which are attributed to the ZIF-90 nanostructure. Furthermore, the SPEES/ZIF-90/3 nanocomposite membrane exhibited exceptional thermal, chemical, and mechanical stability. The excellent proton conductivity of the SPEES/ZIF-90/3 nanocomposite membrane resulted in improved PEMFC performance at 90 °C compared to the standard SPEES membrane. Consequently, the SPEES/ZIF-90/3 nanocomposite membrane emerged as a promising candidate for PEMFC applications. The membrane's superior water uptake and proton conductivity led to superior PEMFC performance, resulting in current densities and power densities of 1.07 A/cm^2^ and 0.52 W/cm^2^, respectively, outperforming the SPEES membrane at 90 °C (Supplementary Information).

## Supplementary Information

Supplementary Information.

## Data Availability

The datasets used and/or analyzed during this paper are publicly available from corresponding author.
