# Supplementary material for: Enhanced performance of nanocomposite membrane developed on sulfonated poly (1, 4-phenylene ether-ether-sulfone) with zeolite imidazole frameworks for fuel cell application
Source: Sci Rep. 2023 May 22;13:8238. doi: 10.1038/s41598-023-34953-8 (PMC10202930; doi:10.1038/s41598-023-34953-8)
Supplement: Supplementary file 1 — Supplementary Information. [file 41598_2023_34953_MOESM1_ESM.docx]

**Supplementary Information**

**Enhanced performance of nanocomposite membrane developed on sulfonated poly (1, 4-phenylene ether-ether-sulfone) with zeolite imidazole frameworks for fuel cell application**

Bita Soleimani^1^, Ali Haghighi Asl^1,🖂^, Behnam Khoshandam^1^, Khadijeh Hooshyari^2^

^1^ Faculty of Chemical, Petroleum and Gas Engineering, Semnan University, Semnan, Iran. ^🖂^email: [ahaghighi@semnan.ac.ir](mailto:ahaghighi@semnan.ac.ir). ^2^ Department of Applied Chemistry, Faculty of Chemistry, Urmia University, Urmia, Iran

Initially, the synthesis of ZIF-90 was meticulously conducted, followed by the subsequent fabrication of the membrane. Subsequent analyses were conducted to validate the characteristics of both the synthesized ZIF-90 and the resulting membranes, which will be elucidated in the following sections. The ZIF-90 nanostructure's successful synthesis was confirmed by FT-IR, XRD, and N_2_ adsorption analyses (Fig. S1, S2 and S3). Microtrac's BELSORP MINI II adsorption instrument measured the Langmuir surface area, specific Brunauer-Emmett-Teller (BET), pore volume, and pore size distribution (Japan). The 8400S model underwent Fourier transform infrared spectroscopy (FTIR). Cu Kα radiation was used in the X-ray diffraction (XRD) analysis, which was performed using the Bruker D8 and GNR Explorer diffractometers (Germany and Italy).

Fig. S1. N_2_ adsorption (filled marks) and desorption isotherm (bank marks) at 77k for ZIF-90.

Fig. S2. XRD pattern of ZIF-90.

Fig. S3. FT-IR spectra of synthesized ZIF-90.

With a resolution of 4 cm^-1^ and a region of 600-4000 cm^-1^, Bruker Equinox 55 was used to perform the ATR-FTIR spectra (Fig. S4). The 8400S model underwent Fourier transform infrared spectroscopy (FTIR). Cu Kα radiation was used in the X-ray diffraction (XRD) analysis, which was performed using the Bruker D8 and GNR Explorer diffractometers (Germany and Italy) (Fig. S5). The morphology of the SPEES/ZIF-90 nanoocomposite membranes was seen using a TESCAN MIRA 3 field emission scanning electron microscope (FESEM) Fig. S6 (a, b). The morphology-phase atomic force microscopy (AFM) JPK NanoWizard II model manufactured by BRUKER was utilized to examine the membrane morphology Fig. S6 (c, d).

Fig. S4. ATR-FTIR spectra of SPEES/ZIF-90/x nanocomposite membrane

Fig. S5. XRD of SPEES/ZIF-90/x nanocomposite membrane


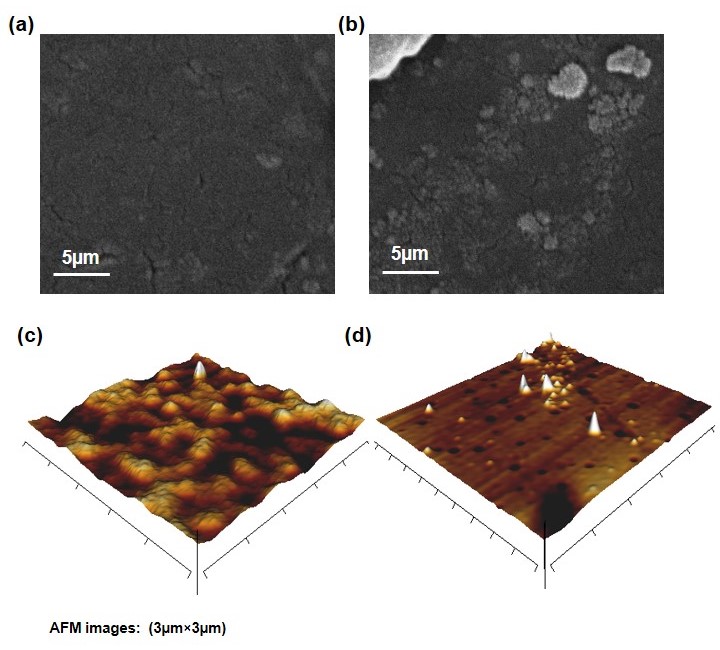


Fig. S6. FESEM-AFM image of cross-section of the (a, c) SPEES/ZIF-90/3, and (b, d) SPEES/ZIF-90/5membranes.

For investigation the oxidation stability of membranes, Fenton test was done based on the procedure explained by Reference ^1, 2^. The weight loss percentage in membrane can be calculated according to:

$\mathrm{WL}\left( \% \right)=(\frac{W0-W1}{W0})\times100$ (1)

Fig. S7. Chemical stability of nanocomposite membranes

Furthermore, we conducted meticulous evaluations of both thermal and mechanical stability tests. For this purpose on a LINSEIS, analyses using thermogravimetric analysis (TGA) were carried out under atmosphere at a heating rate of 10 °C/min (Fig. S8). DSC analyses were obtained using the Q600 (USA) at a rate of 10 °C/min in a N_2_ atmosphere (Fig. S9). Mechanical parameters of the dry membranes were used by Santam STM-50 model with the velocity of 10 mm.min^−1^ (Fig. S10).

Fig. S8. TGA of SPEES, SPEES/ZIF-90/1, SPEES/ZIF-90/3 and SPEES/ZIF-90/5 membranes.

Fig. S9. T_g_ results of SPEES, SPEES/ZIF-90/1, SPEES/ZIF-90/3 and SPEES/ZIF-90/5 membranes.

Fig. S10. Stress-Strain curve.

We conducted comprehensive proton conductivity and fuel cell tests, utilizing the advanced potentiostat-galvanostat Metrohm called the PGSTAT303N. Proton conductivity measurements were meticulously performed using this instrument. The conductivity of protons (σ) was obtained by employing the following relationship ^1^ (Fig. S11. (a, b)):

$\sigma=\frac{L}{RS}$ (2)

Here L represents the membrane thickness (cm), R is the resistance obtained from the Nycost curve (ohm), and S is the membrane surface area (cm^2^).

The slope of the Arrhenius plots can be operated to determine the Activation energy (E_a_) by following relation (3):

$ϭ=A exp(-\frac{E_{a}}{RT})$ (3)

Here, A is the Arrhenius constant, R is gas constant (8.314 J/mol.K) and T was the temperature (Kelvin).

|   **(a)** |   **(b)** |
| --- | --- |

Fig. S11. Proton conductivity of nanocomposite membranes (a) at different temperature and 98% RH, (b) at different temperature 70% RH,

The creation of membrane electrode assemblies (MEAs) is necessary to investigate the PEMFC's final performance. The catalyst ink is first prepared by dissolving the specified quantity of 20 weight percent Pt-C powder in isopropyl alcohol/water and a SPEES solution. A carbon fiber fabric with a microporous layer and a loading of 0.5 mg/cm^2^ will be painted with catalyst ink. The second step involves drying the prepared electrodes between 80 and 120 degrees Celsius. To create the electrode-membrane assembly, the prepared electrodes and membrane were squeezed at 50 kg/cm^2^ for 5 minutes at 120 °C. Finally, the potential was held constant at 0.5 V for 6 hours until the temperature reached 80 °C in order to activate the produced MEAs. Finally at flow rates 300/500 mL/min of hydrogen/Oxygen were inserted into the anode and cathode electrodes. As shown in [Fig.](#fig7) S12 (a, b), the current density-potential (I-V) and current density-power density curves of nanocomposite membranes made of SPEES and SPEES/ZIF-90/3 at 70 °C and 90 °C and 70% RH and 98% RH, respectively. Reporting the OCV of the PEMFC for 100 hours, as shown in [Fig](#fig7) S12 (c), allowed for the determination of the long-term stability of the SPEES/ZIF-90/3 nanocomposite membrane at 90 °C and 98% RH.

**(b)**

**(a)**

|  |  |
| --- | --- |
|  | |

Fig. S12. Polarization curves of SPEES and SPEES/ZIF-90/3 membranes at (a) 70 ^°^C and (b) 90 ^°^C at 70% RH and 98% RH (c) Fuel cell life time plots of SPEES/ZIF-90/3 nanocomposite membranes at 90 ˚C and 98% RH.

**(c)**

The Oxford 600 MHz H-NMR instrument model was employed to analyze the structure of SPEES. Fig. S13 illustrates the H-NMR spectrum of the SPEES polymers. The incorporation of sulfonic acid groups onto the PEES backbone is evidenced by a prominent singlet signal at 7.45 ppm, corresponding to the aromatic proton (He) adjacent to -SO_3_H. Prior to the sulfonation reaction, the He peak was observed at 7.95 ppm for PEES ^1^.


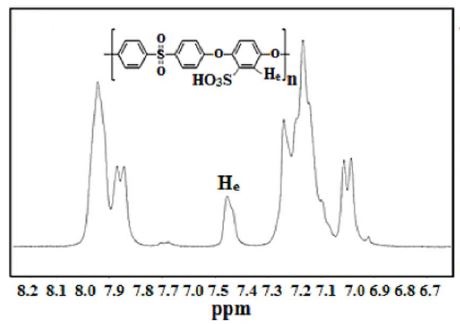


Fig. S13. Plot of the H-NMR of SPEES ^1^.

**Reference:**

1. Hooshyari, K., Khanamiri, S.N., Salarizadeh, P., & Beydaghi, H., Nanocomposite Membranes with High Fuel Cell Performance Based on Sulfonated Poly (1,4-phenylene ether ether sulfone) and Ytterbium/Yttrium Doped-Perovskite Nanoparticles, J. Electrochem. Soc. 166, F976–F989. https://doi.org/10.1149/2.1521912jes (2019).

2. Kim, A.R., Vinothkannan, M., & Yoo, D.J., Sulfonated-fluorinated copolymer blending membranes containing SPEEK for use as the electrolyte in polymer electrolyte fuel cells (PEFC), Int. J. Hydrogen Energy. 42, 4349–4365. https://doi.org/10.1016/j.ijhydene.2016.11.161 (2017).
